# Surface Characterization and Corrosion Behavior of 90/10 Copper-Nickel Alloy in Marine Environment

**DOI:** 10.3390/ma12111869

**Published:** 2019-06-10

**Authors:** Tingzhu Jin, Weifang Zhang, Ning Li, Xuerong Liu, Lu Han, Wei Dai

**Affiliations:** School of Reliability and Systems Engineering, Beihang University, Beijing 100191, China; jintingzhu@buaa.edu.cn (T.J.); zhangweifang@buaa.edu.cn (W.Z.); guess_lining@buaa.edu.cn (N.L.); liuxuerong@buaa.edu.cn (X.L.); 1002154202@cugb.edu.cn (L.H.)

**Keywords:** 90/10 Cu-Ni alloy, marine environment, passive films, surface characterization, corrosion behavior

## Abstract

Surface characterization and corrosion behavior of 90/10 copper-nickel alloy in seawater from Xiamen bay at 30 °C for 56 days were investigated in this study. The results indicated that the corrosion product layer was mainly a mixture of CuO, Cu_2_O, and Cu(OH)_2_, with a transition to CuCl, CuCl_2_, and Cu_2_(OH)_3_Cl during the corrosion process. However, as corrosion proceeds, the resistance of the product film was reduced due to its heterogeneous and fairly porous structures, which led to local corrosion of the alloy. The corrosion potentials (*E_corr_*) increase while corrosion current densities (*I_corr_*) decrease with time because of the formation of protective oxide film.

## 1. Introduction

Copper-nickel alloys are generally known for their good corrosion resistance, excellent machinability, and outstanding thermal and electrical conductivity in marine environments [1,2,3]. The 90/10 copper-nickel alloy has been widely applied in various fields, such as seawater piping, heat exchangers and condensers in ships, desalination plants, power plants, and ship hulls [4,5,6]. The excellent corrosion resistance of Cu-Ni alloy is mainly due to two reasons. First, the ionization of copper is difficult as a result of the positive equilibrium potential and the high thermodynamic stability of Cu. Second, nickel was incorporated into Cu_2_O film and increases the corrosion resistance in two ways: (i) Ni^2+^ occupies the vacant position of Cu^+^ and increase the ionic resistance, which makes two Cu^+^ ions disappear and thus increases the ion resistance of the film; (ii) Ni^2+^ replaces Cu^+^ directly, while the ionic resistance does not change and each substitution results in the disappearance of one Cu^+^ and increases the electronic resistance [7]. Moreover, it is relevant to the formation of a duplex oxide layer on the surface of copper alloy, which is mainly composed of Cu_x_O and Cu-hydroxide/oxide layer and protects the copper matrix. Corrosion products often deposit on the film and play a protective role [4,8,9]. Therefore, copper and copper alloys are more corrosion-resistant than other metal alloys [1].

In marine exposure conditions, Cu_2_O rapidly forms on the surface of the matrix in the initial stage and reacts with chlorides, leading to the formation of CuCl, which usually converts to Cu_2_(OH)_3_Cl as end corrosion products [10,11]. The above components have already been confirmed on bare copper at atmospheric exposures by Fuente [12], and also observed after exposure in laboratory conditions with humidified air and pre-deposited NaCl [13,14]. However, the failure mainly happens on the Cu alloys surface in marine environments and severely disrupts the safe operation of ship and marine engineering [15].

As mentioned in various literatures [16,17,18], it is well-known that the corrosion of Cu-Ni alloys in seawater is a typical electrochemical corrosion and that the main characteristics are: (i) Due to the high chloride ion content (around 19%) in seawater, the Cu-Ni alloy has low anodic polarization and a high corrosion rate in the process of corrosion [2]; (ii) The oxygen depolarization reaction occurs in the Cu-Ni alloy cathode in sea water. Although oxygen is saturated in surface seawater, the rate of oxygen reaching the metal surface through the diffusion layer is less than the rate of cathodic reaction of oxygen reduction. The cathodic process is generally dominated by the diffusion rate of oxygen when the seawater is under stationary state or at low velocity; (iii) The higher conductivity and smaller resistance blockage of seawater corrosion lead to the higher activity of corrosion micro/macro-cells in seawater. Therefore, the main reason for copper corrosion in seawater is that the large amount of Cl^−^ and dissolved oxygen have a great influence on copper and its alloys. At present, many scholars have studied the corrosion behavior of Cu alloys in the NaCl solution, but hav ignored the role of dissolved oxygen [19,20,21]. However, when the oxygen content in seawater is high, the influence of oxygen on copper and its alloys is complex. On the one hand, because the electrode potential of copper is lower than that of oxygen, the oxygen depolarization effect always occurs on the surface of Cu matrix, which makes the corrosion process under cathodic control. On the other hand, oxygen oxidizes cuprous ions Cu^+^ into more corrosive positive copper ions Cu^2+^, which act as oxidant and accelerate copper corrosion. Besides, the oxygen could promote the formation of protective film on the surface of copper. When there are cracks on the surface of Cu and Cu alloys, erosion by Cl^−^ easily destroys the protective film on the surface of the matrix, forming corrosion pits and accelerating the corrosion process of Cu alloys in seawater [22,23]. Besides, there are a large number of mineral ions in seawater, which improve the conductivity of seawater and increase the dissolved oxygen content, all of which provide conditions for copper electrochemical corrosion [24]. Furthermore, in marine environment, high temperature, high salinity, and high dissolved oxygen concentration are the important reasons for serious corrosion of metal materials [25,26,27]. Therefore, it is not rigorous to study the corrosion behavior of metal materials using only artificial corrosion solution in laboratory environment. For example, the corrosion of most metals in marine environment belongs to oxygen depolarization corrosion. With the increase of dissolved oxygen concentration in seawater, the content of oxygen diffusing to metal surface and the speed of oxygen cathode depolarization also increase, which leads to the acceleration of corrosion rate. These results are different from those studied by using NaCl as corrosion solution only [28,29].

Recently, researches on corrosion performance of Cu alloys have been mainly examined in the artificial corrosive solution. Xia et al. [15] have studied the corrosion resistance of the Cu alloy in a 3.5 wt% NaCl solution and demonstrated that the corrosion properties and resistance of Cu-10Ni alloys can be enhanced by ultrasonic surface rolling process (USRP) treatment of surface. Zhu et al. [30] carried out the corrosion behavior of a novel Cu alloy in 3.5% NaCl solution and reported that the oxides and chloride products formed on the surface of the matrix at first and were followed by the formation of dyroxide products. Huang et al. [31] investigated the corrosion behavior of isomorphous Cu-Ni alloy in 3.0% NaCl solution and demonstrated that the corrosion resistance decreased with the Ni concentration increased in the NaCl solution. However, the above researches only paid attention to the influence on the Cl- and neglected other complicated factors in marine environment, such as dissolved oxygen and pH, and were therefore far from realistic service life conditions [32].

Following the above reasons, the objective of this paper is to investigate the surface characterization and corrosion behavior of 90/10 copper-nickel alloy after two months of immersion in the mixed solution of 0.5 mol/L H_2_O_2_ and natural seawater from Xiamen bay in China using XPS, SEM and electrochemistry technique.

## 2. Materials and Methods 

### 2.1. Materials and Corrosion Test

The 90/10 copper-nickel alloy sheet with specifications of 100 mm × 50 mm × 3 mm were studied in the present paper and the composition of the sheet is shown in Table 1.

The surfaces of the specimens were prepared by grinding and polishing with SiC papers and diamond paste sequentially, followed by degreasing in acetone and drying in warm air. The corrosive solution is a mixture of 0.5mol/L H_2_O_2_ and natural seawater from Xiamen bay (24°27′ N latitude, 118°04′ E longitude, and pH 8.2). In order to eliminate the influence of microbiology, the seawater was sterilized by steam at 120 °C for 20 min. The experiment was carried out in a thermostat water bath at 30 °C, while the oxygen was continuously injected into the corrosive solution with an electromagnetic charge pump.

### 2.2. Characterization of the Corrosion Product Film

#### 2.2.1. Morphology Observation Analysis by SEM/EDS

After corrosion in the mix solution, a set of specimens were cleaned with deionized water and then dried in air. Another set of samples was immersed in sulfuric acid solution diluted 10 times for 3 min, of which relative density was 1.84, and then removed the corrosion products by hard brush. The prepared specimens were treated by spray gold, SEM with EDS (CS3400, CamScan, Nottingham, UK) were used to study the microstructure.

#### 2.2.2. Raman Spectroscopy Analysis

The Raman spectroscopy was analyzed to recognize the oxidation state of Cu element on the surface at room temperature by Maya2000 Pro-NIR spectrometer (Ocean Optics, Oxford, UK), and a He-Ne laser with 785 nm line was used to exciting the specimens. The surfaces of the alloy after corrosion for several days in backscattering configuration were examined with a 50-fold objective lens. Spectral measurements were recorded from 100 to 1200 cm^−1^ at different points on the surface for each specimen.

#### 2.2.3. XPS Analysis

To further detect the composition at different depths of the corrosion surface, the XPS of the prepared specimens was carried out by K-Alpha X-ray photoelectron spectrometer (thermo escalab 250, Thermo Fisher Scientific, Gloucester, UK). Prior to XPS test, the specimens were treated in deionized water and dried using a vacuum oven [33]. The Al Ka X-ray source were used to motivate the high-resolution photoelectron of Cu2p and O1s, while the output data were analyzed by the XPS PEAK 1 software (Version 4.1).

### 2.3. Electrochemical Measurement

The corrosion behavior of the samples immersed for 0 d, 7 d, 14 d, 28 d, and 56 d was characterized by potentiodynamic polarization curves and was determined using a CHI660D workstation (Huachen, Shanghai, China) in the corrosive solution at 20 ± 1 °C with a conventional three-electrode cell. The solutions were in contact to air to get saturated with oxygen, before the electrochemical measurements, while after the measurements, the electrolyte is no longer aerated. The potentiodynamic polarization curves were measured in the range from −0.4 V to 0.0 V (vs. SCE) of a scan rate of 0.01 mV/s the samples.

## 3. Results and Discussion

### 3.1. XPS Analysis of the Corrosion Product Layer

#### 3.1.1. Cu2p Spectra

**Figure 1:**
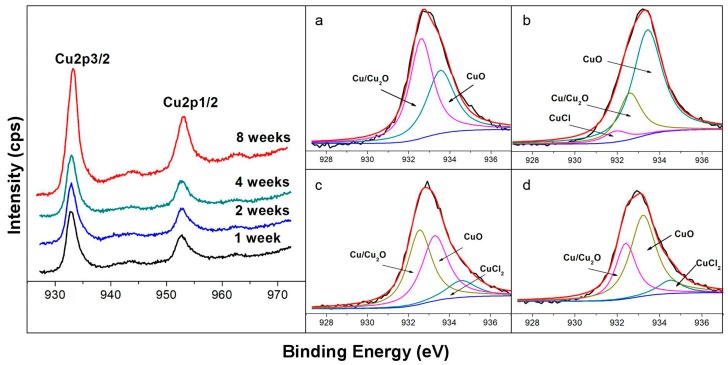
Cu XPS spectra of the corrosion product layer on the surface of the 90/10 Cu-Ni alloy in the natural seawater for different days: (**a**) Core-level Cu2p3/2 spectrum for 7 days; (**b**) core-level Cu2p3/2 spectrum for 14 days; (**c**) core-level Cu2p3/2 spectrum for 28 days; (**d**) core-level Cu2p3/2 spectrum for 56 days.

Figure 1 shows the high-resolution Cu2p spectra (at 925 eV–975 eV) on the corrosion product layer of the prepared samples after exposure in the seawater for 7 days, 14 days, 28 days, and 56 days, respectively. It is noted that the oxide film grows up with the extension time would present a positive shift of binding energy (B.E.) on Cu2p spectra [1]. According to the high resolution of Cu2p spectra in Figure 1, the peaks of Cu2p3/2 and Cu2p1/2 are appearing at 932.5 eV and 952.5 eV, while the core-level of the Cu2p3/2 spectra is presented at B.E. from 928eV to 938 eV as shown in Figure 1a–d, respectively. Besides, the spectrum exhibited the shake-up features around at 943.5 eV and 962 eV for Cu2p3/2 and Cu2p1/2 peaks, respectively, which indicate that CuO is dominant after corrosion due to the presence of the unfilled (d9) valence level of the Cu^2+^ ion, and other forms of Cu may exist, such as Cu_2_O and Cu(OH)_2_ [34,35].

The relative quantities in Figure 1 are shown in Table 2. It is demonstrated that the primary components of the product film are CuO and Cu_2_O with the prolonging exposure time from 7 days to 56 days. It is difficult to distinguish the peaks of metallic Cu and Cu_2_O due to the similar binding energy, so they are expressed as Cu/Cu_2_O [33]. After exposure to seawater for seven days, as displayed in Figure 1a, two peaks at the B.E. of 933.5eV and 932.6eV are presented in the deconvolution of the core-level Cu2p3/2, and it can be seen that Cu species were composed of 43.2% CuO and 56.8% Cu/Cu_2_O as listed in Table 2 [36,37]. Figure 1b shows 3 peaks at B.E. of 933.5, 931.9 and 932.5 eV in the deconvolution of core-level Cu2p3/2 spectrum after corrosion for 14 days. According to Table 2, it is believed that the constituent of the corrosion product film is 69.6% CuO, 6.1% CuCl, and 24.3% Cu/Cu_2_O after corrosion for 14 days [36,37,38]. The results indicated that the primary constituents of the product film are CuO and Cu_2_O, while a small amount of CuCl was also existed in the layer. It should be pointed out that the formation of a porous CuCl is mainly identified as the first stage in the corrosion process of Cu alloys [2,17,39]. With a prolonged exposure time of 28 days, as shown in Figure 1c and Table 2, there are three deconvoluted peaks of core-level Cu2p3/2 spectrum at B.E. of 934.5, 933.5, and 932.5 eV, which are corresponded to CuCl_2_, CuO, and Cu/Cu_2_O [36,37,38,39,40]. It should be noted that CuCl disappeared and a small amount of CuCl_2_ (12.6%) was detected instead. Furthermore, the relative quantity of Cu_2_O increased significantly from 24.3% to 44.7%, while the CuO decreased from 69.6% to 42.7% after 28 days of exposure. These indicated that a cuprous oxide layer (Cu_2_O) and a cupric oxide layer (CuO) formed the duplex corrosion product layer on the alloy surface [1]. In the later stage of corrosion, the core-level Cu2p3/2 spectra for 56 days exhibited three peaks at B.E. of 934.5, 933.5, and 932.4 eV, which is due to CuCl_2_, CuO, and Cu/Cu_2_O, as shown in Figure 1d [36,37,38,39,40]. Besides, according to the relative quantity of Cu2p3/2 spectra, two obvious features can be discerned: (i) The relative amount of CuO (58.4%) and Cu_2_O (32.4%) were major in Cu2p3/2 spectra over the corrosion period, despite the Cu_2_O decreased from 44.7% to 32.4%; (ii) a small amount of CuCl_2_ (9.2%) was also detectable in the Cu2p spectra.

#### 3.1.2. O1s spectra

**Figure 2:**
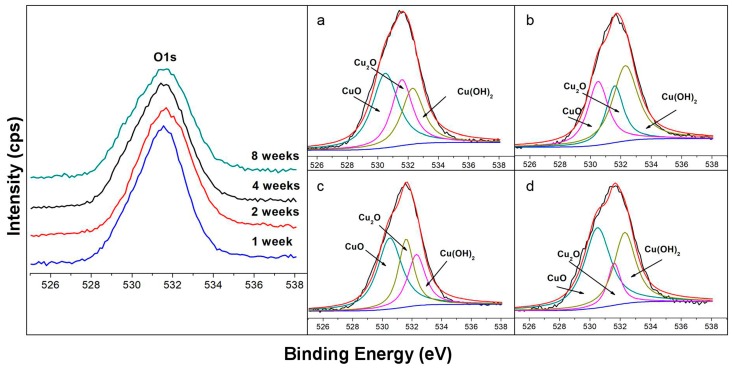
O XPS spectra of the corrosion product layer on the surface of the 90/10 Cu-Ni alloy in the natural seawater for different days: (**a**) Core-level O1s spectrum for 7 days; (**b**) core-level O1s spectrum for 14 days; (**c**) core-level O1s spectrum for 28 days; (**d**) core-level O1s spectrum for 56 days.

The O1s spectra are considered together with the spectra of Cu2p to predict the possible corrosion products [41]. Figure 2 shows the O1s spectra (at 525 eV–538 eV) of the corrosion product film of the samples surface immersed in the seawater for 7 days to 56 days. The core-level spectrum presents three peaks at B.E. of 530.5, 531.6, and 532.3 eV in the high-resolution O1s spectra. It should be noted that, due to the existence of Ni element in Cu-Ni alloy and for the same type of oxides, their binding energy was probably the same. Thus, the peaks at the B.E. of 530.5, 531.6, and 532.3 eV, as listed in Table 3, could be identified as CuO/NiO, Cu_2_O, and Cu(OH)_2_/Ni(OH)_2_ [2,41].

As given in Table 3, at the early stage of corrosion (seven days), the main component in the corrosion product layer is presented as CuO (43%). With the immersion time extending to 14 days, Cu(OH)_2_/Ni(OH)_2_ (45%) became more dominant than CuO (31%), while at longer exposure times (56 dasy), the relative quantity of CuO increased significantly from 31.0% to 50.4%, while the Cu(OH)_2_/Ni(OH)_2_ decreased from 45.2% to 36.3% after 56 days of exposure. These results are generally consistent with Cu2p spectra. However, the difference is that the Cu(OH)_2_ did not appear in the Cu2p spectra, and this phenomenon is in accordance with the literature by Xiao [33]. They considered that the intensity of the Cu(OH)_2_ in Cu2p spectra is pretty weak [33], while the combination of Cu(OH)_2_ and Ni(OH)_2_ lead to the result in O1s spectra.

The current XPS study demonstrates that the corrosion of 90/10 Cu-Ni alloy exposed in marine environment contains the cathodic (reduction of O_2_) and anodic reaction (oxidation of Cu) as follows [2,41]:

(1) $$O_{2}+{2H}_{2}O+{4e}^{-}\to {4\mathrm{OH}}^{-},$$

(2) $$\mathrm{Cu}+{\mathrm{Cl}}^{-}\to \mathrm{CuCl}+e^{-},$$

The CuCl always generates on the alloy surface and forms the loose CuCl film due to the low solubility [44]. Along with the corrosion time prolonging, the CuCl adsorption film dissolves gradually and forms the copper complex of CuCl_2_^−^, as follows:

(3) $$\mathrm{CuCl}+{\mathrm{Cl}}^{-}\to {\mathrm{CuCl}}_{2}^{-},$$

Besides, the ${\mathrm{CuCl}}_{2}^{-}$ would react with OH^−^ to form the cuprous Cu_2_O film, which would reduce the corrosion rate of the Cu alloy in seawater, as follows [1]:

(4) $${2\mathrm{CuCl}}_{2}^{-}+{2\mathrm{OH}}^{-}\to \mathrm{Cu}2O+H_{2}O+{4\mathrm{Cl}}^{-},$$

In the marine environment, there is a large amount of dissolved oxygen as described earlier in the “Introduction” section. Carley et al [45] pointed out that the formation of a Cu(II) state on the surface of Cu alloy was due to the existence of adsorbed oxygen. With long exposure time, Cu_2_O hydrolysis generated Cu(OH)_2_ as follows [1,46]:

(5) $${\mathrm{Cu}}_{2}O+3H_{2}O\to {2\mathrm{Cu}(\mathrm{OH})}_{2}+{2H}^{+}+2e^{-}$$

### 3.2. Raman Analysis of the Corrosion Product Layer

**Figure 3:**
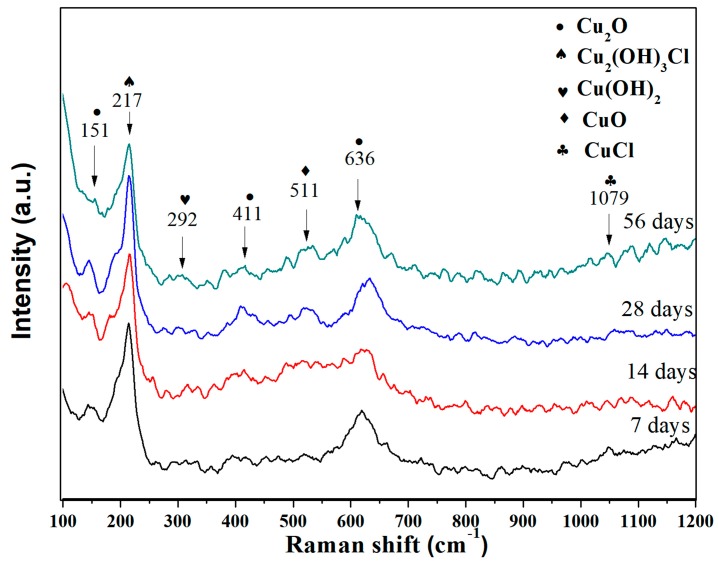
Raman spectrum of the corrosion product film formed on the surface of the 90/10 Cu-Ni alloy exposed in seawater for 28 days.

The Raman spectrum was investigated of the alloy surface with several corrosion days for better identified the structures of the product film, as illustrated in Figure 3. Each sample was tested three times by Raman spectroscopy to confirm the accuracy. The presence of Cu_2_O (peaks at 151 cm^−1^ and 411 cm^−1^ [47]), CuO (peak at 511 cm^−1^ [48]), CuCl (peak at 1079 cm^−1^ [48]), and Cu(OH)_2_ (peak at 292 cm^−1^ [47]) are detected in the corrosion product, which is in accordance with the XPS results. However, the Cu_2_(OH)_3_Cl (peak at 217 cm^−1^ [48]) is also found in Raman measurement, while it was not detected in the XPS spectra, demonstrating that just a small amount of Cu_2_(OH)_3_Cl exists on the corrosion surface [3].

### 3.3. Macrostructure Analysis

**Figure 4:**
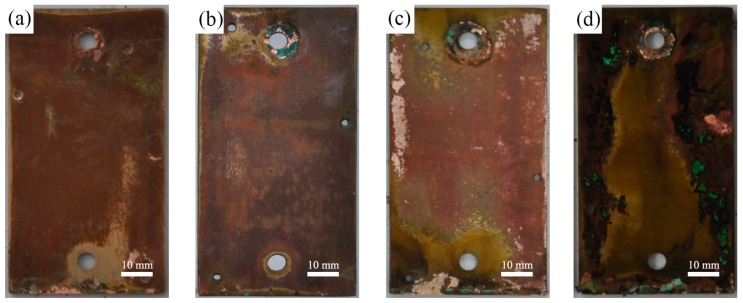
The macroscopic surface morphologies of Cu-Ni alloy after corrosion for (**a**) 7 d, (**b**) 14 d, (**c**) 28 d, and (**d**) 56 d (before removing the corrosion products).

The macroscopic morphologies of the specimens corroded for 7 d, 14 d, 28 d, and 56 d are shown in Figure 4. The red corrosive products were presented obviously on the surface of the alloy after immersed for 7 d, as carried out in Figure 4a, which were considered as Cu oxidations (Cu_2_O), as mentioned by Qin et al. [43,49]. With the corrosion time prolonging, the massive red corrosion products were accumulated, some yellow corrosion products were formed on the alloy surface, and the color turned darker, as presented in Figure 4b,c. After a longer corrosion time (immersion for 56 d), the whole surface turned brown and piled up some green corrosion products, as displayed in Figure 4d.

**Figure 5:**
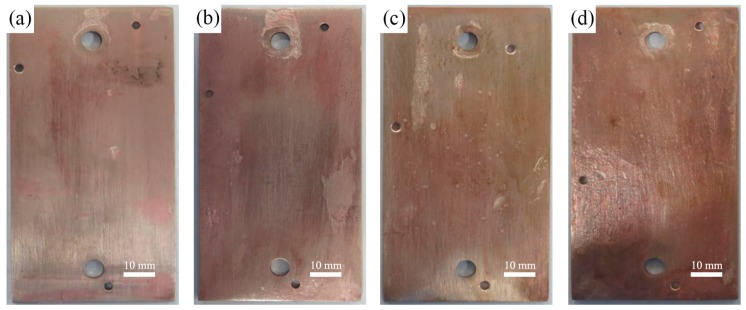
The macroscopic surface morphologies of Cu-Ni alloy after corrosion for (**a**) 7 d, (**b**) 14 d, (**c**) 28 d, and (**d**) 56 d (after removing the corrosion products).

The surface morphologies of the sample after removing the corrosion products were shown in Figure 5. It is revealed that the surface of the sample showed slight corrosion pits after corrosion for seven days, and the corrosion area gradually increased with the extension of time. With the time prolonging to 56 days, the corrosion area increased significantly, and the surface of the sample was exfoliated.

Generally speaking, the corrosion products of B10 Cu-Ni alloy are multilayer [50]. The inner layer film is a kind of uniformly adhered brick red corrosion product, and often turns into a black and membranous cuprous oxide layer. Due to the superposition of red and black oxide layers, the protective film on the corrosion of Cu alloys are often yellow-brown in appearance. In the presence of Cl^−^ in seawater, due to the combined action of Cl^−^ and dissolved oxygen in seawater, the red/black oxide product film is partially damaged, which leads to the deepening of the local corrosion of the Cu matrix. The green and loose corrosion products are often formed when the serious corrosion occurs and in form of piles or patches.

### 3.4. Microstructure Analysis

**Figure 6:**
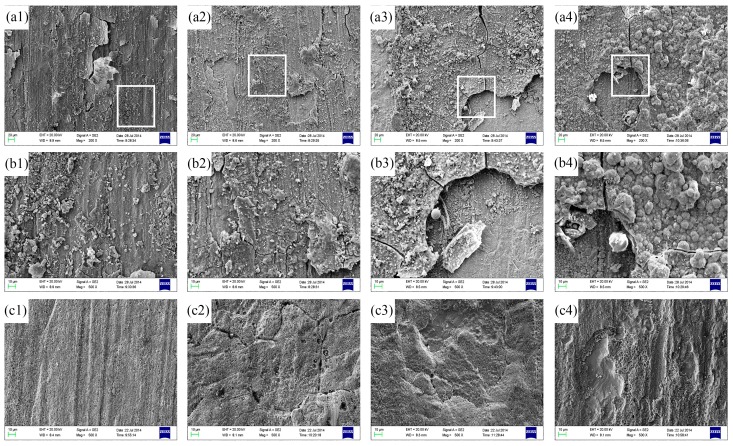
SEM micrographs of specimen surface after different corrosion time before (**a1**–**a4** and **b1**–**b4**) and after (**c1**–**c4**) removing corrosion products: (**a1**,**b1**) 7 d, (**a2**,**b2**) 14 d, (**a3**,**b3**) 28 d, (**a4**,**b4**) 56 d, (**b**) the magnified image of the white box in (**a**); (**c1**) 7 d, (**c2**) 14 d, (**c3**) 28 d, (**c4**) 56 d.

The surface morphologies and composition of the corrosion product films deposited on the alloy matrix were studied by the use of SEM and EDS energy (dispersive X-ray spectrometry) analysis, as shown in Figure 6 and Table 4.

Figure 6a1–a4 show the SEM micrographs of surface with corrosion product on the samples exposed for several days, while Figure 6b1–b4 shows magnified images of the white box in Figure 6a1–a4. The EDS analyses of the whole area in Figure 6b1–b4 are shown in Table 4. The corresponding microstructure of the surface after removing corrosion products are shown in Figure 6c1–c4.

After corrosion for seven days, as shown in Figure 6a1,b1, the pitting areas are small and the Cu matrix is still visible. Besides, some white and grey products gradually appeared on the surface. According to the EDS results, the product film was, perhaps, a combination of the cupric oxide film (CuO and Cu(OH)_2_) and cuprous oxide (Cu_2_O) [1]. The corresponding microstructure of the surface after removing corrosion products are shown in Figure 6c1, it is shown that the pores initiate from the specimen surface with white corrosion products after corrosion for seven days, which was in agreement with the Reference [51]. After being corroded for 14 days, the pitting area increased significantly and lots of corrosion products piled up on the surface, as shown in Figure 6a2,b2. The EDS revealed the presence of copper and oxygen, which were likely present as CuO and Cu_2_O. Besides, it is indicated that the typical intergranular corrosion appeared on the alloy surface after pickling as demonstrated in Figure 6c2, which was in agreement with the Reference [52]. When testing time up to 28 days, as shown in Figure 6a3,b3, much more particle-like corrosion products deposited on the surface and some products fall off from the sample because of the inadhesive structure and stresses [53]. The EDS analysis indicated that the corrosion products mainly consisted of the oxide and chlorine, and the results are also in accordance with literature [3,8]. According to the microstructure after pickling, the exfoliation corrosion presented on the surface as shown in Figure 6c4. With the extension corrosion time to 56 days, plenty of particle-like corrosion products accumulated and almost fully cover the entire surface of the alloy, as observed in Figure 6a4,b4. Furthermore, there was obvious speckle corrosion in the Figure 6c4, and exfoliation also occurred locally.

The results demonstrate that the corrosion product films on the surface are heterogeneous and fairly porous, as well as all present cracks and exfoliation, which are due to the compactness, high fragility, poor cohesion, and adhesion [12]. Generally speaking, the corrosion of the sample first started from the grain boundary. With the prolongation of immersion time, corrosion continues to expand along grain boundaries, and gradually develops to the surface of the whole sample, forming copper oxide film. At the beginning of film formation, the film is not uniform and the adsorption of Cl^−^ is also inconsistent. The nickel corrosion occurs on the surface of the sample, resulting in poor protective performance of the film in some areas. The Cu_2_O film is divided into inner layer and outer layer [54], in which the outer layer is produced by the deposition of dissolved Cu^2+^, while the inner layer is produced by the more compact and protective Cu_2_O film. The grain boundaries, twins, or other defects on the Cu matrix are under the corrosion product film. The growth rate of the corrosion product film in these areas is slower than that in the intergranular due to the difference of structure and composition. As the corrosion time was prolonged, the oxidation products in the outer layer formed on the surface and the content gradually increased. However, the strength of the corrosion product film is lower than its internal stress, so the products are porous and easy to fall off from the sample, which led to cracks in the film [50].

### 3.5. Polarization Curve Measurements

**Figure 7:**
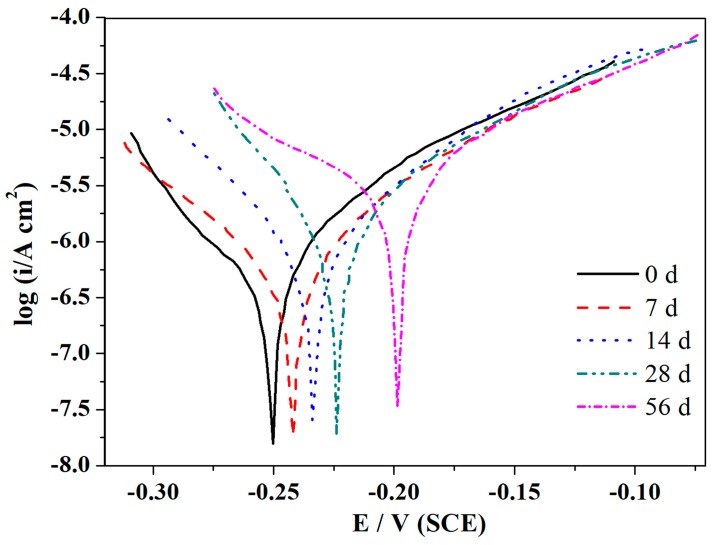
The polarization curves of Cu-Ni alloy after being exposed in corrosion solution for different days.

Figure 7 demonstrates the polarization curves of the 90/10 Cu-Ni alloy after being exposed for several days. The corresponding values calculated from the polarization curves, such as anodic and cathodic Tafel slope (*β_a_* and *β_c_*, respectively), corrosion potentials (*E_corr_*), and corrosion current densities (*I_corr_*), are given in Table 5. The resistance of the polarization *R_p_* can be calculated as follows:

(6) $$R_{p}=\frac{\beta_{a}\times \left|\beta_{c}\right|}{2.3(\beta_{a}+\beta_{c})\times I_{corr}},$$

It can be observed that the *E_corr_* shift about 50 mV to positive direction with the extension corrosion time to 56 days, which was in virtue of the inhibitive effect of protective oxide film and corrosion on the Cu surface as a result of decreasing the rate of the anodic reaction [55]. Besides, the *I_corr_* decreased quickly from 3.812 to 1.056 μA/cm^2^ after 56 days of corrosion. This were mainly due to the generation of a protective oxide film and the reduction of the Cl^−^ attack on the alloy surface, which resulted in the reduction of corrosion rate and the homogeneous corrosion of Cu alloy. In addition, the increasing *β_a_* was concerned with the decreasing corrosion reaction caused by the reduction of anodic currents. This is identical with the experiment results of Zhu Xiao in studying the corrosion behavior of a novel Cu alloy in a salt spray environment [34].

Generally, in the initial stage of corrosion, the surface of Cu matrix is relatively smooth, while the halogen ions, dissolved oxygen, and corrosion products are free to diffuse on the surface of the matrix, so the corrosion of the matrix surface is controlled by activation. Cu is oxidized to CuO in the passivation zone, while Cu_2_O is oxidized in the activation zone, and chloride is formed by reacting with Cl^−^ in marine environment. Cu_2_O has thermodynamic instability and is easy to be oxidized into copper ions. With the increasing immersion time, the corrosion product film gradually forms on the surface of the matrix and the Cu_2_O film became thicker, so the combination of protective oxide film and corrosion product film inhibits the diffusion of halogen ions, dissolved oxygen, and corrosion products. At this point, the anode reaction is dominated by the mass transfer process, while the cathode process remains relatively unchanged, which will lead to an increase in corrosion potential. Studies [55,56] have shown that the electronic properties of the surface film are related to its composition and structure, so that the surface film shows different types of semiconductors in different potential ranges. Generally speaking, Cu_2_O is a typical P-type semiconductor, while high valence oxides, such as Ni and Fe, are N-type semiconductors. This bipolar structure can play a good protective role. For the B10 alloy, in the range below the flat-band potential, the space charge layer of Ni and Fe oxides in the surface film is in the state of enrichment and acts as the conductor, while the space charge layer of the Cu oxide part is depleted and shows a P-type semiconductor. On the contrary, in the range higher than the flat potential, the space charge layer of Cu oxide part in the surface film is in the enrichment state, which is equivalent to the conductor, while the space charge layer of Ni and Fe oxide is depleted and behave N-type semiconductor. According to the point defect model, the corrosion product film contains various high concentration point defects, such as metal ion vacancy and oxygen vacancy. In marine environment, on the one hand, the Cu_2_O on the surface of the film may occur redox transformation of copper oxide (CuO) or copper hydroxide (Cu(OH)_2_). On the other hand, when the corrosion product film contacts with Cl^−^, the oxygen vacancies in the N-type semiconductor on the outer surface of the surface film would adsorb Cl^−^ in solution and Mott-SchottkyPair reaction would occur with Cl^−^ to produce oxygen/metal ion vacancy pairs. The oxygen vacancies can also generate more metal ion vacancies with other Cl^−^ reactions. Thus, more and more metal vacancies accumulate at the metal matrix/film interface, isolating the matrix from the surface film and preventing the film from continuing to grow. Furthermore, due to the increased potential as mentioned above, Cl^−^ adsorbed on the surface film and resulted in the thickened Cu_2_O film, which formed a porous green corrosion product, namely, cupric chloridehydroxide (Cu_2_(OH)_3_Cl) [50]. When the product layer grows to the inner film, the dynamic equilibrium of the dissolution of the outer layer is destroyed, causing the local tension of the product film to exceed the theoretical breaking strength, resulting in penetrating rupture of the surface film. Therefore, the greater the concentration of defects (oxygen vacancy and metal ion vacancy) in the surface film is, the more vulnerable the surface coating is to erosive ions. As corrosion proceeds, continuous Cl^−^ penetrates into the defects of the CuO film and reduces the resistance of the protective film, resulting in localized collapse, and pittings are formed on the surface of the substrate and expanded with the growth of the microvoids under the aggressive attack of Cl^−^ [42]. At this time, the dissolution of copper produces new corrosion active sites, which hinders the further formation of protective film and leads to more serious local corrosion on the surface.

## 4. Conclusions

Surface characterization and corrosion behavior of 90/10 copper-nickel alloy immersed in seawater from Xiamen bay at 30 ℃ for 56 days were investigated using XPS, SEM analyses, and electrochemical measurements. According to the results, following conclusions can be summarized:

1. The corrosion product formed on the surface of the 90/10 Cu-Ni alloy mainly contained CuO, Cu_2_O and Cu(OH)_2_. With increasing exposure time, CuCl, CuCl_2_, and Cu_2_(OH)_3_Cl were also formed on the surface, which to some extent protected the alloy from corrosion.
2. The corrosion product films on the surface are heterogeneous and fairly porous, as well as all present cracks and exfoliation, which are due to the compactness, high fragility, poor cohesion, and adhesion. More serious local corrosion would occur in the 90/10 Cu-Ni alloy during the process of seawater corrosion.
3. Electrochemical results showed that, with the extension of exposure time, the *E_corr_* shifted to positive direction, while the *I_corr_* shifted to negative, which were due to the inhibitive effect of protective oxide film and corrosion on the Cu surface as a result of decreasing the rate of the anodic reaction and the Cl^−^ attack on the surface.

## Figures and Tables

**Table 1:** Composition (wt%) of the experimental 90/10 copper-nickel alloy sheet.

Ni	Fe	Mn	Zn	P	Si	S	Cu
9.76	1.2	0.74	<0.3	<0.02	<0.15	0.0013	Bal.

**Table 2:** Fitting parameters for Cu 2p3/2 XPS spectra and relative quantity of compounds in the corrosion layers of 90/10 Cu-Ni alloy exposed to seawater for different days.

Valence State	Exposure Time (Days)	Proposed Compounds	Binding Energy (eV)	Intensity Area	Relative Quantity	FWHM ^1^ (eV)	Ref.
Cu 2p3/2	7	CuO	933.5	3918.2	0.432	1.617	[37]
		Cu/Cu_2_O	932.6	5146.3	0.568	1.341	[36]
	14	CuO	933.5	13637.2	0.696	1.642	[37]
		CuCl	931.9	1193.7	0.061	1.376	[38]
		Cu/Cu_2_O	932.5	4753.6	0.243	1.289	[37]
	28	CuCl_2_	934.5	1190.4	0.126	1.734	[40]
		CuO	933.5	4050.1	0.427	1.518	[37]
		Cu/Cu_2_O	932.5	4231.2	0.447	1.385	[36]
	56	CuCl_2_	934.5	850.1	0.092	1.367	[40]
		CuO	933.5	5380.5	0.584	1.428	[37]
		Cu/Cu_2_O	932.4	2976.8	0.324	1.146	[36]

^1^ FWHM: Full Width Half Maximum.

**Table 3:** Fitting parameters for O1s XPS spectra and relative quantity of compounds in the corrosion layers of 90/10 Cu-Ni alloy exposed to seawater for different days.

Valence State	Exposure Time (Days)	Proposed Compounds	Binding Energy (eV)	Intensity Area	Relative Quantity	FWHM (eV)	Ref.
O1s	7	CuO	530.5	12042.5	0.430	2.048	[41]
		Cu_2_O	531.6	8369.92	0.299	1.609	[42]
		Ni(OH)_2_/Cu(OH)_2_	532.3	7581.14	0.271	1.720	[43]
	14	CuO	530.5	8524.94	0.310	1.592	[41]
		Cu_2_O	531.6	6526.19	0.238	1.364	[42]
		Ni(OH)_2_/Cu(OH)_2_	532.3	12395.8	0.452	1.957	[43]
	28	CuO	530.5	13184.5	0.469	1.948	[41]
		Cu_2_O	531.6	7977.35	0.284	1.234	[42]
		Ni(OH)_2_/Cu(OH)_2_	532.3	6964.84	0.247	1.429	[43]
	56	CuO	530.5	12647.8	0.504	2.06	[41]
		Cu_2_O	531.6	3352.98	0.133	1.035	[42]
		Ni(OH)_2_/Cu(OH)_2_	532.3	9109.82	0.363	1.674	[43]

**Table 4:** The EDS analysis of specimen surface composition in Figure 6b1–b4.

Corrosion Time (d)	Overall Film Composition (at%)				
	Cu	Ni	O	Cl	Others
7	44.15	6.06	44.08	2.02	3.69
14	36.46	6.19	47.80	7.01	2.54
28	30.78	7.33	49.37	8.26	4.26
56	30.39	5.29	51.53	8.45	4.34

**Table 5:** Characteristic parameters obtained from the polarization curves of the two alloys after being exposed in corrosion solution for different days.

Exposure Time (Day)	*β_a_* (mV/decade)	*β_c_* (mV/decade)	*E_corr_* (V)	*I_corr_* (μA/cm^2^)	*R_p_* (KΩ/cm^2^)
0	52.3	−212.3	−0.249	3.812	4.786
7	49.5	−222.7	−0.241	1.310	13.488
14	51.2	−228.6	−0.234	1.284	14.165
28	53.5	−286.6	−0.224	1.129	17.359
56	65.71	−183.32	−0.198	1.056	19.916
